# Daily physical activity in ankylosing spondylitis: validity and reliability of the IPAQ and SQUASH and the relation with clinical assessments

**DOI:** 10.1186/ar4279

**Published:** 2013-08-23

**Authors:** Suzanne Arends, Marianne Hofman, Yvo PT Kamsma, Eveline van der Veer, Pieternella M Houtman, Cees GM Kallenberg, Anneke Spoorenberg, Elisabeth Brouwer

**Affiliations:** 1 Rheumatology and Clinical Immunology, University of Groningen, University Medical Center Groningen, P.O. Box 30.001, 9700 RB Groningen, the Netherlands; 2 Rheumatology, Medical Center Leeuwarden, P.O. Box 888, 8901 BR Leeuwarden, the Netherlands; 3 Center for Human Movement Sciences, University of Groningen, University Medical Center Groningen, P.O. Box 30.001, 9700 RB Groningen, the Netherlands; 4 Laboratory Medicine, University of Groningen, University Medical Center Groningen, P.O. Box 30.001, 9700 RB Groningen, the Netherlands

## Abstract

**Introduction:**

The aim of this study was to investigate the construct validity and test-retest reliability of the International Physical Activity Questionnaire (IPAQ; long form) and the Short QUestionnaire to Assess Health-enhancing physical activity (SQUASH) and to investigate the relation between daily physical activity and clinical assessments in patients with ankylosing spondylitis (AS).

**Methods:**

For validity, the self-report questionnaires IPAQ and SQUASH were compared with daily physical activity assessed with the ActiGraph accelerometer during 7 consecutive days in 63 AS outpatients. For reliability, the IPAQ and SQUASH were administered twice approximately 1 week apart in 52 AS outpatients. In all 115 patients, clinical assessments were performed at the outpatient clinic.

**Results:**

IPAQ and SQUASH total scores correlated significantly with accelerometer outcome: ρ = 0.38 and *r *= 0.35, respectively. Intraclass correlation coefficients between first and second assessments of the IPAQ and SQUASH were 0.83 and 0.89, respectively. Bland-Altman analyses showed no systemic bias, but in particular for the IPAQ the 95% limits of agreement were wide. Daily physical activity assessed by accelerometer, IPAQ, and SQUASH correlated significantly with disease activity, physical activity, and quality of life. A relation with spinal mobility was found only for the accelerometer and SQUASH. The direction of these correlations indicates that higher daily physical activity is related to lower disease activity and better physical function, spinal mobility and quality of life.

**Conclusions:**

Both physical activity questionnaires showed modest construct validity. The SQUASH showed good test-retest reliability, superior to the IPAQ. These results indicate that the SQUASH is more suitable than the IPAQ to assess daily physical activity in AS population studies. However, it is desirable to add questions on AS-specific physical activity. Further studies are needed to investigate the causality of the relation between daily physical activity and clinical assessments.

## Introduction

Ankylosing spondylitis (AS) is a chronic, progressive, inflammatory disease that primarily affects the axial skeleton. The disease causes pain and reduced spinal mobility, which can lead to limitations in physical functioning. AS usually starts from early adult age up to the fourth decade of life [1]. Consequently, AS patients can become limited in their professional and leisure-time activities at a relatively early age.

Besides pharmacological treatment, exercise and physical therapy are considered essential components of treatment [2]. A Cochrane review on exercise programs and physical therapy revealed small but beneficial effects on physical function, spinal mobility, and patient global assessments in AS [3]. Data concerning the relation between the total amount of daily physical activity (that is, household, work, transport, and leisure-time activities) and clinical assessments are scarce. Recently, Haglund and colleagues showed that lower disease activity, better physical function, and better health-related quality of life were positively associated with meeting the World Health Organization global recommendations of physical activity for health in a heterogeneous group of patients with spondyloarthritis, including AS [4].

Physical activity is a complex and multidimensional exposure variable, which makes population-based measurement difficult [5]. Multiple measurement techniques are used to quantify physical activity. These techniques can be subdivided into two categories: direct methods such as stable isotopes to assess total energy expenditure (doubly labeled water) and accelerometers or pedometers, and indirect methods such as oxygen uptake or heart rate monitoring and questionnaires or logs. Physical activity questionnaires are considered the most applicable method for population-based studies because of participant convenience and minimal cost [5-8].

Recently, van Poppel and colleagues recommended that questionnaires assessing total physical activity should at least measure duration and frequency, and should cover physical activity in all settings (household, work, transport, recreation, and sport) in order to reach sufficient content validity [9]. The International Physical Activity Questionnaire (IPAQ) and the Short QUestionnaire to Assess Health-enhancing physical activity (SQUASH) are recall questionnaires that fulfill these recommendations. Both questionnaires have acceptable construct validity and moderate to high test-retest reliability in healthy populations [10,11]. To our knowledge, no studies have been published concerning the measurement properties of physical activity questionnaires in AS.

The aim of the present study was to investigate the construct validity and test-retest reliability of the IPAQ long form and the SQUASH in patients with AS. The second aim was to investigate the relation between daily physical activity and clinical assessments of disease activity, physical function, spinal mobility, and quality of life in these patients.

## Patients and methods

### Patients

Between March 2010 and May 2011, 115 consecutive AS outpatients from the Medical Center Leeuwarden (*n *= 63) and the University Medical Center Groningen (*n *= 52) were included. All patients were over 18 years of age and fulfilled the modified New York criteria for AS [12] or the Assessments in Ankylosing Spondylitis (ASAS) criteria for axial spondyloarthritis including magnetic resonance imaging [13]. Patients with concomitant conditions restricting physical activity or patients not able to read Dutch were excluded. The study was approved by the local ethics committees of the Medical Center Leeuwarden and University Medical Center Groningen, and written informed consent according to the Declaration of Helsinki was obtained from all patients.

### Construct validity

Construct validity of the self-reported physical activity questionnaires was examined by correlating the IPAQ and SQUASH total scores to the accelerometer outcome. The accelerometer provides an objective and valid estimate of overall physical activity [6]. On the first day, the long version of the IPAQ and the SQUASH were administered in randomized order at the outpatient clinic in 63 AS patients. In succession, daily physical activity was assessed using the ActiGraph accelerometer during 7 consecutive days (days 2 to 8).

#### International Physical Activity Questionnaire

The IPAQ long form refers to an average week in the past month and comprises questions in the following domains: occupational, household and gardening, transport, and leisure time [10]. Each of these domains provides a specific activity score calculated by multiplying the number of minutes per week of the performed activities with the accompanying mean metabolic equivalent (MET) value of these activities [14,15]. The total activity score was calculated by the sum of these domain scores and was reported in MET-minutes/week. According to the IPAQ guidelines, data were excluded if the total minutes of activity per day exceeded 960 minutes or when values were missing [10].

#### Short QUestionnaire to Assess Health-enhancing physical activity

The SQUASH refers to an average week in the past month and contains questions in the following domains: commuting activities, household activities, leisure-time and sports activities, and activities at work and school. Activity scores per domain were calculated by multiplying the number of minutes per week with an intensity score (range 1 to 9) of the activities performed [11]. The intensity score was based on the reported intensity of an activity combined with the activity intensity classification according to Ainsworth's Compendium of Physical Activities [14,15]. The total activity score was calculated as the sum of the scores per domain. According to the SQUASH protocol, data were excluded if the total minutes of activity per day exceeded 960 minutes [11]. Since the present study investigates the validity and reliability of the SQUASH, data were also excluded if values were missing.

#### Accelerometer

The ActiGraph accelerometer (GT1M; MTI, Fort Walton Beach, FL, USA) is a small, lightweight, uniaxial accelerometer, which senses vertical accelerations between 0.05 and 2.0 gravitational acceleration with a sample frequency of once per minute. The ActiGraph accelerometer is the only commercially available accelerometer that has repeatedly been shown to correlate with doubly labeled water-derived energy expenditure [16], which is considered the gold standard.

The accelerometer was worn on the right hip during waking hours, except for periods of showering or other water activities. The outcome of the accelerometer was expressed in average kilo counts per day (kcounts/day), calculated by dividing the total activity kilocounts by the total number of days the monitor was worn. Data were excluded if the accelerometer was worn for less than 10 hours per day, for less than 5 days, or when it was not worn during both weekend days [10,17].

Owing to the uniaxial measurement property of the ActiGraph accelerometer, measuring cycling and fitness activities (particularly strength training) is limited. The ActiGraph cannot be used in the water and is therefore not capable of measuring swimming or other water activities. Because of these limitations, the accelerometer outcome was also correlated with an adjusted total score of the SQUASH excluding questions related to cycling, fitness, and swimming.

### Test-retest reliability

The test-retest reliability of an instrument is based on the assumption that the construct has not changed over time and thus the outcome of the measure can be reproduced. To test the reproducibility of the IPAQ and the SQUASH over time, both questionnaires were filled out in randomized order by 52 AS patients on two different occasions approximately 1 week apart (median 8 days, range 5 to 31). This time period was chosen because it is unlikely that the disease status will change in a week and this period is long enough to avoid recall bias. The first assessment was performed at the outpatient clinic and the second assessment was conducted at home.

### Clinical assessments

In all 115 patients, clinical assessments were performed at the outpatient clinic on the first day of the questionnaire assessments. Disease activity was assessed using the Bath Ankylosing Spondylitis Disease Activity Index (BASDAI; on a scale of 0 = no disease activity to10 = worst possible disease activity), the erythrocyte sedimentation rate, C-reactive protein (CRP), and the Ankylosing Spondylitis Disease Activity Score (ASDAS_CRP_; calculated from BASDAI Questions 2, 3, and 6, patient's global assessment of disease activity, and CRP - a higher score represents higher disease activity) [18,19]. Physical function was assessed using the Bath Ankylosing Spondylitis Functional Index (BASFI; on a scale of 0 = no functional limitations to 10 = worst possible physical function). Spinal mobility assessments included occiput-to-wall distance, chest expansion, modified Schober test, lateral spinal flexion (mean of left and right) and cervical rotation (mean of left and right). Higher scores for these assessments represent better spinal mobility, except for occiput-to wall distance. Quality of life was assessed using the Ankylosing Spondylitis Quality of Life questionnaire (ASQoL; on a scale of 0 = best possible quality of life to 18 = worst possible quality of life).

### Statistical analysis

Data were analyzed using PASW Statistics 18 (SPSS, Chicago, IL, USA). Construct validity was examined by calculating Spearman's and Pearson's correlation coefficients between accelerometer activity counts and IPAQ and SQUASH total scores, respectively. Correlations below 0.3 were interpreted as poor association, between 0.3 and 0.6 as modest association, between 0.6 and 0.8 as good association, and above 0.8 as excellent association. A *z *test with Fisher's transformation was used to compare the correlation between accelerometer outcome and the SQUASH total score with the correlation between accelerometer outcome and an adjusted SQUASH total score without cycling, fitness, and swimming.

Test-retest reliability of the IPAQ and the SQUASH was investigated by calculating intraclass correlation coefficients (ICCs; two-way random-effects model, single measures, absolute agreement) between the first and the second assessments of the questionnaires. Reliability was assessed for both total scores and activity scores per domain. ICC values of at least 0.70 indicate good reliability [20]. Additionally, Bland-Altman analysis was performed on the total scores [21].

Pearson's and Spearman's correlation coefficients were used as appropriate to analyze the relation between daily physical activity and clinical assessments. Multivariate linear regression analysis was used to correct these relations for age, gender, and seasonal variation. Since the IPAQ total score was non-normally distributed, this parameter was log-transformed before being entered into the equation. *P *< 0.05 were considered statistically significant.

## Results

Characteristics of the 115 AS patients included in the validity (*n *= 63) and reliability (*n *= 52) studies are presented in Table 1.

**Table 1:** Characteristics of the ankylosing spondylitis study population

	Total group	Validity study	Reliability study
Number of patients	115	63	52
Age (years)	44.6 ± 12.1	43.2 ± 12.3	46.3 ± 11.8
Gender (male)	71 (62)	40 (64)	31 (60)
Body mass index	26.4 ± 4.4	26.2 ± 4.8	26.6 ± 3.9
Duration of symptoms (years)	16 (0 to 54)	18 (2 to 54)	16 (0 to 53)
Time since diagnosis	10 (0 to 42)	11 (1 to 42)	9 (0 to 37)
HLA-B27^+^	82 (76)	49 (82)	33 (69)
Anti-TNF use	78 (70)	39 (62)	39 (81)*
NSAID use	44 (40)	24 (39)	20 (42)
DMARD use	17 (15)	13 (21)	4 (8)
BASDAI (range 0 to 10)	3.7 (0.0 to 9.0)	3.8 (0.4 to 8.6)	3.5 (0.0 to 9.0)
ESR (mm/hour)	11 (2 to 60)	13 (2 to 60)	10 (2 to 39)
CRP (mg/l)	3 (2 to 46)	3 (2 to 44)	4 (2 to 46)
ASDAS_CRP_	2.3 (0.7 to 4.4)	2.3 (0.9 to 4.2)	2.3 (0.7 to 4.4)
BASFI (range 0 to 10)	3.8 ± 2.4	3.6 ± 2.4	4.2 ± 2.4
Occiput-to-wall distance (cm)	0.0 (0.0 to 29.0)	0.0 (0.0 to 29.0)	0.0 (0.0 to 20.0)
Chest expansion (cm)	5.0 (1.0 to 14.0)	6.0 (1.0 to 10.5)	4.0 (1.0 to 14.0)**
Modified Schober test (cm)	4.0 (0.4 to 7.0)	4.0 (0.4 to 6.2)	3.9 (0.0 to 7.0)
Lateral spinal flexion (cm)	10.0 (0.0 to 27.0)	10.0 (0.0 to 25.0)	10.0 (1.8 to 27.0)
Cervical rotation (degrees)	63 (5 to 98)	70 (7 to 95)	58 (5 to 98)
ASQoL (range 0 to 18)	6 (0 to 18)	6 (0 to 17)	6 (0 to 18)

Data presented as mean ± standard deviation, *n *(%) or median (range). ASDAS, Ankylosing Spondylitis Disease Activity Score; ASQoL, Ankylosing Spondylitis Quality of Life; BASDAI, Bath Ankylosing Spondylitis Disease Activity Index; BASFI, Bath Ankylosing Spondylitis Functional Index; CRP, C-reactive protein; DMARD, disease-modifying antirheumatic drug; ESR, erythrocyte sedimentation rate; HLA-B27^+^, human leukocyte antigen B27-positive; NSAID, nonsteroidal anti-inflammatory drug; TNF, tumor necrosis factor. *Statistically significant difference compared to the validity study (*P *< 0.05). **Statistically significant difference compared to the validity study (*P *< 0.01).

### Completeness of data

Complete data to examine the validity of the IPAQ and the SQUASH were available for 45 (71%) and 53 (84%) of the 63 patients, respectively. In these patients, the median IPAQ total score was 5,937 MET-minutes/week (interquartile range 2,126 to 11,601), the mean SQUASH total score was 7,267 (standard deviation ± 3,453), the mean accelerometer outcome was 236 kcounts/day (standard deviation ± 106), and the mean wear time of the accelerometer was 14.3 hours/day (standard deviation ± 1.8). The remaining patients had missing IPAQ values (*n *= 12), had missing (*n *= 2) or invalid (*n *= 1) SQUASH values, and/or did not meet the accelerometer criteria (*n *= 7). Patient characteristics were comparable for patients with and without complete data.

Complete data for the first and second assessments of the IPAQ and the SQUASH were available for 32 (62%) and 33 (63%) of the 52 patients, respectively. The remaining patients had missing IPAQ values (*n *= 18), had missing SQUASH values (*n *= 16), the second SQUASH questionnaire was missing (*n *= 1), or the total minutes of accelerometer activity per day exceeded 960 minutes (*n *= 2).

Patients with incomplete IPAQ data were older (*P *< 0.01), had longer symptom duration (*P *< 0.01), and had worse spinal mobility (occiput-to-wall distance (*P *< 0.01) and chest expansion (*P *< 0.05)). Patient characteristics were comparable for patients with and without complete SQUASH data.

### Construct validity

**Figure 1:**
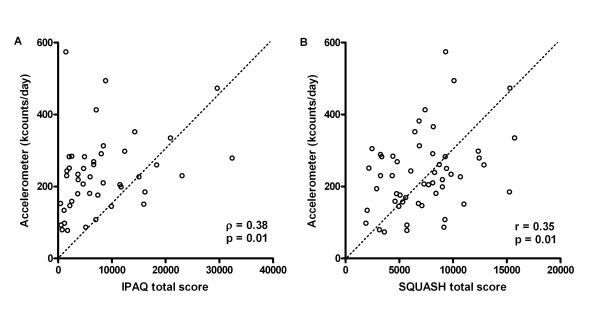
**Relation between questionnaire total scores and accelerometer activity counts**. Relation between **(A) **International Physical Activity Questionnaire (IPAQ) and **(B) **Short QUestionnaire to Assess Health-enhancing physical activity (SQUASH) total scores and accelerometer activity counts.

The correlation between the IPAQ total score and accelerometer activity counts was 0.38, (*P *< 0.05). The correlation between the SQUASH total score and accelerometer activity counts was 0.35 (*P *< 0.05). These values indicate modest validity of the IPAQ and the SQUASH (Figure 1).

According to the SQUASH, 68%, 34%, and 19% of AS patients reported cycling, fitness (health club exercise), and swimming, respectively, which are activities that cannot be (properly) registered by the accelerometer. The correlation between the SQUASH total score adjusted for these activities and accelerometer outcome was 0.39 (*P *< 0.01). This correlation did not significantly differ from the correlation with the unadjusted SQUASH total score (*z *= -0.228, *P *= 0.82).

### Test-retest reliability

The ICC for the IPAQ total score was 0.83, indicating good reliability. ICCs for the subscores ranged from 0.60 (household and garden activities) to 0.88 (transport activities). The ICC for the SQUASH total score was 0.89, representing good reliability. ICCs for subscores of the SQUASH ranged from 0.48 (commuting activities) to 0.88 (work and school activities) (Table 2).

**Table 2:** Physical activity of ankylosing spondylitis patients (with complete data) included in the reliability study

	First assessment	Second assessment	ICC	95% confidence interval
IPAQ (MET-minutes/week) (*n *= 32)				
Total activity score	3,849 (1,470 to 8,132)	4,349 (2,120 to 7,508)	0.83*	0.68 to 0.91
Work activity score	0 (0 to 3,710)	50 (0 to 3,197)	0.80*	0.63 to 0.90
Transport activity score	653 (284 to 1,607)	737 (209 to 1,589)	0.88*	0.77 to 0.94
Household and garden activity score	580 (53 to 1,481)	245 (90 to 1,406)	0.60*	0.33 to 0.79
Leisure time activity score	657 (195 to 1,718)	678 (111 to 1,832)	0.73*	0.52 to 0.86
SQUASH (*n *= 33)				
Total activity score^a^	5,760 (3,360 to 6,890)	5,600 (3,715 to 7,540)	0.89*	0.79 to 0.95
Work and school activity score	1,080 (0 to 4,440)	600 (0 to 4,680)	0.88*	0.77 to 0.94
Commuting activity score	0 (0 to 341)	0 (0 to 438)	0.48*	0.17 to 0.70
Household activity score	900 (420 to 1,815)	840 (240 to 1,635)	0.77*	0.59 to 0.88
Leisure-time and sports activity score	1,350 (775 to 1,795)	1,410 (480 to 2,635)	0.87*	0.76 to 0.93

Data are presented as median (interquartile range). No significant differences were found between the first and second assessments of the physical activity questionnaires. ICC, intraclass correlation; IPAQ, International Physical Activity Questionnaire; MET, metabolic equivalent; SQUASH, Short QUestionnaire to Assess Health-enhancing physical activity. ^a^SQUASH total activity score was normally distributed with mean 5,617 (standard deviation ± 3,233) and 5,808 (standard deviation ± 3,825) at the first and second assessment, respectively. *Statistically significant intraclass correlation (*P *< 0.001).

**Figure 2:**
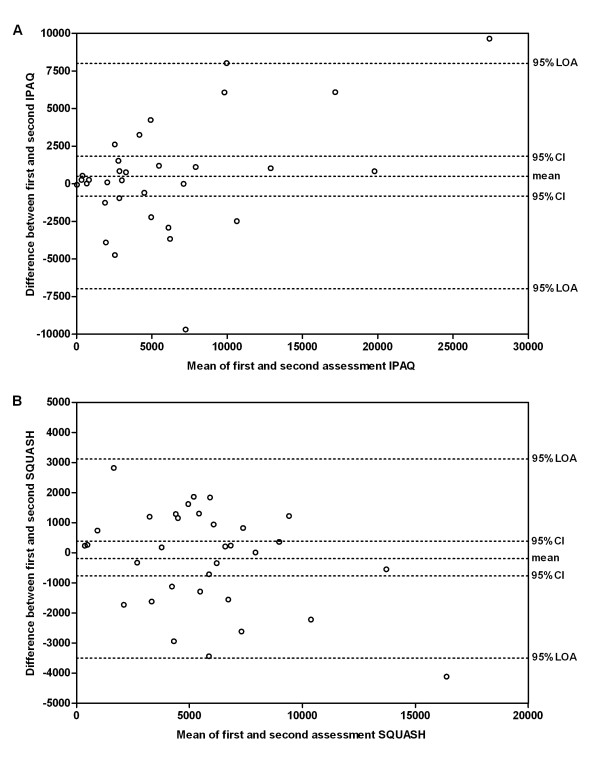
**Bland-Altman plots**. Difference between the total scores on the first and second **(A) **International Physical Activity Questionnaire (IPAQ) and **(B) **Short QUestionnaire to Assess Health-enhancing physical activity (SQUASH) plotted against the mean of both assessments, together with 95% limits of agreement (LOA). CI, confidence interval.

Bland-Altman analyses for the IPAQ and the SQUASH total scores showed that the mean difference between the first and second assessments was small and not significantly different from zero, which implies that no systematic bias was present. However, in particular for the IPAQ, the 95% limits of agreement (LOA) were wide, meaning that only large changes can be considered true changes (Figure 2).

### Relation between daily physical activity and clinical assessments

Accelerometer, IPAQ and SQUASH total scores correlated significantly with disease activity assessed using the ASDAS_CRP_. In addition, the IPAQ and the SQUASH correlated significantly with the BASDAI, and accelerometer scores correlated significantly with the erythrocyte sedimentation rate and CRP. All three daily physical activity measures correlated significantly with physical function and quality of life, while a significant correlation with spinal mobility was found only for the accelerometer and the SQUASH (Table 3). The direction of these correlations indicates that higher daily physical activity is related to lower disease activity and better physical function, spinal mobility and quality of life.

**Table 3:** Correlations between daily physical activity and clinical assessments in patients with ankylosing spondylitis

	IPAQ (MET-minutes/week)	SQUASH	Accelerometer (kcounts/day)
Number of patients	86^a^	94^a^	55^b^
Disease activity			
BASDAI (range 0 to 10)^c^	-0.220*^d^	-0.326**^d^	NS
ESR (mm/hour)^c^	NS	NS	-0.460***^d^
CRP (mg/l)^c^	NS	NS	-0.289*^d^
ASDAS_CRP_^c^	-0.243*^d^	-0.311**^d^	-0.283*^d^
Physical function			
BASFI (range 0 to 10)^c^	-0.387***^d^	-0.476***^e^	-0.274*^e^
Spinal mobility			
Occiput-to-wall distance (cm)^c^	NS	-0.297**^d^	NS
Chest expansion (cm)^f^	NS	NS	NS
Modified Schober test (cm)^f^	NS	0.260*^d^	0.338*^d^
Lateral spinal flexion (cm)^f^	NS	NS	0.369*^e^
Cervical rotation (degrees)^f^	NS	0.306**^d^	0.320**^d^
Quality of life			
ASQoL^c^	-0.282**^d^	-0.500***^d^	-0.356**^d^

ASDAS, ankylosing spondylitis disease activity score; ASQoL, ankylosing spondylitis quality of life; BASDAI, Bath ankylosing spondylitis disease activity index; BASFI, Bath ankylosing spondylitis functional index; CRP, C-reactive protein; ESR, erythrocyte sedimentation rate; IPAQ, International Physical Activity Questionnaire; MET, metabolic equivalent; NS, nonsignificant; SQUASH, Short QUestionnaire to Assess Health-enhancing physical activity. ^a^Only patients with complete data on the first IPAQ or SQUASH assessment and available clinical data were included. ^b^Only patients with complete accelerometer data and available clinical data were included. ^c^Lower score represents lower disease activity or better physical function, spinal mobility or quality of life.^d^Spearman's correlation coefficient. ^e^Pearson's correlation coefficient. ^f^Higher score represents better spinal mobility. *Statistically significant correlation (*P *< 0.05). **Statistically significant correlation (*P *< 0.01). ***Statistically significant correlation (*P *< 0.001).

Multivariate linear regression analysis showed that all of the above-mentioned correlations remained statistically significant after correcting for age, gender, and seasonal variation, except for the correlation between accelerometer outcome and the ASDAS_CRP _(*P *= 0.108).

## Discussion

This is the first study that investigates the measurement properties of physical activity questionnaires in AS. The construct validity of the IPAQ (long form) and the SQUASH compared with accelerometer activity counts was found to be modest, with correlations of 0.38 and 0.35, respectively. A large study in healthy populations from different countries showed comparable agreement between the IPAQ total score and accelerometer outcome (pooled correlation of 0.33) [10]. The results for the SQUASH total score were reported to be somewhat better, with correlations of 0.45 in healthy adults and 0.67 in patients after total hip arthroplasty [11,22]. Terwee and colleagues have recently developed the Quality Assessment of Physical Activity Questionnaire checklist to appraise the qualitative attributes and measurement properties of physical activity questionnaires. They stated that the correlation between total physical activity assessed by a questionnaire and accelerometer total counts should be at least 0.50 [23]. Based on these guidelines, the standard to prove the construct validity of the IPAQ and the SQUASH was not reached in patients with AS.

Both questionnaires showed good test-retest reliability based on ICC values (0.83 and 0.89 for the IPAQ and SQUASH total scores, respectively). Previous studies reported Spearman's correlation coefficients of 0.81 (pooled correlation) for the IPAQ long form [9] and 0.58 and 0.57 for the SQUASH [11,22]. In the present study, ICCs were used instead of correlation coefficients because correlation coefficients do not take systematic measurement errors into account [23]. For comparison with previous studies, correlation coefficients were also calculated, resulting in values of 0.74 for the IPAQ and 0.90 for the SQUASH (data not shown). Bland-Altman analysis was performed in only one of the previous studies. In accordance with our results, the authors reported no systematic bias between SQUASH assessments, but their 95% LOA were approximately three times larger than those found in the present study [22]. We found wide 95% LOA for the IPAQ in comparison with the SQUASH, which indicates that only large changes can be considered true changes.

Interestingly, objective accelerometer daily activity was significantly correlated with the objective measures erythrocyte sedimentation rate and CRP, and the subjective IPAQ and SQUASH scores were significantly correlated with the subjective measure BASDAI. Furthermore, the IPAQ and SQUASH scores were significantly correlated to the ASDAS_CRP_, a composite score of patient-reported measures and CRP developed in order to capture both subjective and objective aspects of AS disease activity. The inverse relation between accelerometer outcome and CRP is in line with recent findings of Plasqui and colleagues [24]. The correlation between accelerometer outcome and ASDAS_CRP _did not remain significant after correcting for age, gender, and seasonal variation. This can probably mainly be explained by the patient-reported aspects of the ASDAS, since also no significant correlation was found between accelerometer outcome and the BASDAI.

Besides the relation with disease activity, we also found significant relations with physical function, spinal mobility, and quality of life. These findings are in accordance with Haglund and colleagues, who found that lower disease activity (BASDAI), better physical function (BASFI), and better health-related quality of life (EuroQoL) were positively associated with meeting the World Health Organization recommendations of moderate-intensity and/or vigorous-intensity physical activity in a heterogeneous group of spondyloarthritis patients [4]. Some additional support for these relationships can be obtained from intervention studies concerning exercise programs and physical therapy in AS. Although exercise is only part of a person's total physical activity [25], an increase in exercise means in general an increase in total physical activity. Until now, conflicting data have been published about the relation between physical activity and disease activity in AS intervention studies [26,27]. Randomized controlled trials showed an improvement in physical function and spinal mobility after an 8-week to 4-month exercise program [28-30]. Further studies are needed to investigate the causality of this relation between higher daily physical activity and better clinical assessments.

A limitation of this study was the large amount of missing values in both questionnaires. A relatively large group of patients therefore had to be excluded from the analyses. The frequent occurrence of missing values may hamper the use of these physical activity questionnaires in daily practice. Patients with incomplete IPAQ data were significantly older and had longer symptom duration and worse spinal mobility, indicating that this questionnaire may be less suitable for patients with advanced AS. These findings refer to a limitation in feasibility. Patient characteristics were comparable for patients with and without complete SQUASH data. In contrast to the IPAQ, the SQUASH guidelines clearly prescribe how to deal with missing data [9]. However, the reliability of the SQUASH total score was insufficient (ICC = 0.60) in our analysis including patients with imputation for missing values according to the SQUASH guidelines (data not shown).

A second limitation is that the GT1M ActiGraph accelerometer used in this study is a uniaxial accelerometer and therefore is limited in the measurement of physical activities that require little vertical movement, such as cycling or fitness (especially strength training). In addition, the accelerometer is not waterproof and therefore swimming and other water-related activities could not be measured. Despite this, the correlation between the accelerometer outcome and the SQUASH score without these activities was comparable with the correlation of the total SQUASH score. The IPAQ only discriminates between moderate and vigorous leisure-time physical activities. For that reason, a correction for cycling, fitness, and swimming on the IPAQ total score could not be performed.

Finally, the timeframes for the questionnaires and accelerometer were not completely comparable. Both questionnaires referred to a usual week in the past month (retrospective assessment), whereas the accelerometer daily activity was assessed prospectively for 5 to 7 days. Biological variation in physical activity can thus have decreased the correlations between the IPAQ or the SQUASH and accelerometer outcome. Wearing the accelerometer may make the patients more aware of their physical activity level. An advantage of our method is therefore that accelerometry could not influence the physical activity questionnaire assessments. Furthermore, wearing the accelerometer could not influence the reliability assessments of the questionnaires, since validity and reliability were examined in two different groups of AS patients.

The main difference between the IPAQ and the SQUASH is that the IPAQ measures walking, moderate, and vigorous activities, whereas the SQUASH measures all activities (light, moderate, and vigorous). The domains of both questionnaires are approximately the same, but the sports domain of the SQUASH is more detailed. In the IPAQ, the activity score is based only on the MET value. In the SQUASH, the MET value is related to the age of the participant and combined with the self-reported intensity to calculate the activity score. Overall, the SQUASH measures total daily physical activity in more detail and more straightforwardly than the IPAQ, which probably resulted in the better performance of the SQUASH in this study.

Disease-specific physiotherapy and exercise programs are important in the management of AS. However, no specific question about this topic is included in the SQUASH (or IPAQ) and not all patients reported these activities in the section about leisure-time and sport activities. Therefore, it is desirable to include AS-specific questions in the SQUASH. The drawback of adapting the questionnaire, however, is that it would no longer be possible to compare the results with studies in the general population or in other diseases. The best solution is probably to develop questions for AS-specific exercises in addition to the existing SQUASH questions.

## Conclusions

In the present study, the construct validity of the IPAQ (long form) and the SQUASH was found to be modest in patients with AS. Both questionnaires showed good test-retest reliability based on ICC values, and Bland-Altman analyses showed no systemic bias between assessments. In particular for the IPAQ, the 95% LOA were wide, which indicates that the degree of repeatability is insufficient. Moreover, analysis of missing data revealed that the IPAQ may be less suitable for patients with advanced AS. Daily physical activity assessed by accelerometer outcome (objective) and IPAQ and SQUASH total scores (subjective) was found to be significantly related to clinical assessments of disease activity, physical function, and quality of life. A relation with spinal mobility was found only for the accelerometer and the SQUASH.

Based on these results, the SQUASH seems superior to the IPAQ to assess daily physical activity in AS population studies. However, it is desirable to add questions on AS-specific physical activity. Further studies are needed to investigate the causality of the relation between better clinical assessments and higher daily physical activity in AS.

## Abbreviations

AS: ankylosing spondylitis; ASDAS: Ankylosing Spondylitis Disease Activity Score; BASDAI: Bath Ankylosing Spondylitis Disease Activity Index; CRP: C-reactive protein; ICC: intraclass correlation coefficient; IPAQ: International Physical Activity Questionnaire; LOA: limits of agreement; MET: metabolic equivalent; SQUASH: Short QUestionnaire to Assess Health-enhancing physical activity.

## Competing interests

The authors declare that they have no competing interests.

## Authors' contributions

SA and MH participated in the design of the study, performed the statistical analysis and interpretation of data, performed the acquisition of physical activity data, and drafted the manuscript. YPTK and EvdV participated in the design of the study, contributed to the interpretation of data, and critically revised the manuscript. PMH contributed to the acquisition of clinical data and critically revised the manuscript. CGMK contributed to the design of the study and critically revised the manuscript. AS and EB participated in the design of the study, performed the acquisition of clinical data, and critically revised the manuscript; All authors approved the final manuscript.
